# Depression, Social Engagement, and Cognitive Function: A Mediation Analysis

**DOI:** 10.1093/geroni/igaf122.2986

**Published:** 2025-12-31

**Authors:** Yuchen Gu

**Affiliations:** Boston College, Brighton, Massachusetts, United States

## Abstract

Cognitive abilities are essential for older persons to maintain functional independence. Previous studies have shown that depression is linked to cognitive decline and a higher risk of dementia, while increased social engagement is associated with better cognitive performance. Since depressive disorders are frequently coexisted with severe and extensive social isolation, it is possible that social engagement is a mediator between depression levels and cognitive function. Nevertheless, there is no standardized measurement of social engagement. Whereas some researchers operationalize social engagement as a composite scale of various activities, others examine distinct forms of social engagement based on various theories. I examine the mediating effect of social, physical, and cognitive activities categorized by exploratory factor analysis. Using the KHB decomposition method from waves 14 (2018) and 15 (2020) of the Health and Retirement Study (HRS), I investigate how three different types of social activities can mediate the association between depression levels and cognition among older persons aged 65 and over (n = 1,896). This study finds that depression is negatively associated with cognitive function, and social, physical, and cognitive activities each partially mediate this relationship. Additionally, social activity has the strongest mediating effect, followed by physical and cognitive activities. These findings suggest that future interventions on promoting older people’s social engagement could be a potential strategy to mitigate the negative effects of depression on cognitive function.

